# Preliminary Research on the Health-Promoting Value of Honeydew Honey Enriched with Bee Bread

**DOI:** 10.3390/molecules30020256

**Published:** 2025-01-10

**Authors:** Alicja Sęk, Sara Olszak, Katarzyna Jaśkiewicz, Teresa Szczęsna

**Affiliations:** The National Institute of Horticultural Research, Konstytucji 3 Maja 1/3, 96-100 Skierniewice, Polandkatarzyna.jaskiewicz@inhort.pl (K.J.); teresa.szczesna@inhort.pl (T.S.)

**Keywords:** honey, perga, bee product mixtures, oxidative stress, antioxidants, phenolic compounds

## Abstract

Since the imbalance between free radicals and antioxidants in the body plays a significant role in the physiology of common, often dangerous diseases, an emphasis is placed on enriching the daily diet with compounds characterized by antioxidant activity. Good sources of natural antioxidants are bee products such as honey, bee pollen, bee bread and propolis, and the best path for introducing the latter products into the diet is mixing them with honey. However, the characteristics of bee product mixtures are not yet fully understood. Therefore, the aim of this study is to verify the health-promoting properties of a mixture of honeydew honey and multifloral bee bread. The profile of phenolic compounds, radical scavenging activity, total phenolic content, diastase number, and also proline and HMF content were determined. The obtained results indicated the improved health-promoting value of this mixture, as increases in radical scavenging activity (from 82.7 to 88.4%), in the total content of phenolic compounds (from 74.6 to 118.8 mg·100 g^−1^), and also in the proline content (from 64.0 to 95.5 mg·100 g^−1^) and diastase activity (from 22.6 to 38.8 Schade units) were observed when 5% of bee bread (*w*/*w*) was added. Moreover, the bee bread addition provided two important flavonoids to the honeydew honey, i.e., rutin and kaempferol.

## 1. Introduction

Oxidative stress, defined as a disturbance in the balance between free radicals and antioxidants in the body, plays an active role in the physiology of common diseases such as diabetes, high blood pressure, pre-eclampsia, atherosclerosis, acute renal failure, Alzheimer’s and Parkinson’s disease [1]. It is also thought to be involved in the development of cancer, autism and depression [2,3,4]. An important action to prevent and control the problem of oxidative stress is to invest in research into new products that will be effective in free radical scavenging. In recent years, many studies have focused on the antioxidant potential of honey, as its usage in the treatment of various diseases has been reported [5,6]. The high antioxidant activity of honey is related to the presence of phenolic acids, flavonoids and other antioxidants including amino acids, organic acids, ascorbic acid, glucose oxidase, catalase, carotenoid derivatives and proteins in its chemical composition [7]. However, it should be remembered that the specific chemical profile of honey is variable and depends on its botanical and geographical origin [8].

Among the many varietal honeys, manuka honey seems to be the most promising so far; however, knowledge about its physicochemical properties, particularly after it is imported from the New Zealand area, is limited. Very recently, we unveiled a low diastase number (DN), i.e., lower than 8 Schade units which is the minimum required for honey of good quality, in the manuka honey available on the Polish market [9]. The low diastase activity was accompanied by a relatively high hydroxymethylfurfural (HMF) content, which demonstrated the flawed physicochemical quality of these commercially available honeys, as both a low DN and a high HMF content may result from long-term storage, storage in improper conditions, or thermal treatment [9]. This encourages further research into bee products, and perhaps mixtures of bee products, in order to obtain a product with the most beneficial functional properties.

The literature shows that dark honeys, such as honeydew honeys, are superior to light ones in terms of antioxidant properties, which is directly due to their higher antioxidant content [10,11]. Bee pollen and bee bread are also promising sources of antioxidants [12,13]. Among the two, bee bread is more nutritious since the fermentation process renders the final product more digestible and enriched with additional biomolecules [14]. Moreover, two new studies indicate a positive effect of bee pollen and bee bread addition on the antioxidant properties of multifloral honey [15,16]. It has also been proven that bee bread, when used alone, in addition to its antioxidant, antibacterial, antifungal and antiviral activities, has an effective glycemia-lowering effect [17,18,19]. Thus, in the present study, we addressed the issue of the health-promoting properties of honeydew honey enriched with bee bread. We focused our attention on the analysis of the profile of phenolic compounds, activity against the DPPH radical, total phenolic content, DN and proline and HMF content in the absence and presence of bee bread in honeydew honey samples obtained from the Island Beskid mountain range (Poland).

## 2. Results and Discussion

The first stage of research included confirmation of the variety of honey in usage and a melissopalynological analysis of the examined bee bread. A conductometric analysis showed that the electrical conductivity of the tested honey sample was 1.32 ± 0.07 mS·cm^−1^, and therefore, according to the European Directive, the honey can be classified as honeydew honey [20]. The obtained result is consistent with the existing literature which shows that the electrical conductivity of Polish honeydew honeys is in the range of 0.96–1.32 mS·cm^−1^ [21]. It is worth mentioning at this point that electrical conductivity measurement is a routine method used to distinguish nectar honey from honeydew honey [22]. Honeydew honeys are generally darker in color, while darker honeys contain higher levels of minerals and organic acids, and thus they indicate higher values of electrical conductivity (>0.8 mS·cm^−1^) [23]. In turn, the pollen analysis indicated that the bee bread can be classified as multifloral, since no dominance of any pollen type was found in the sample (see Table 1).

**Figure 1:**
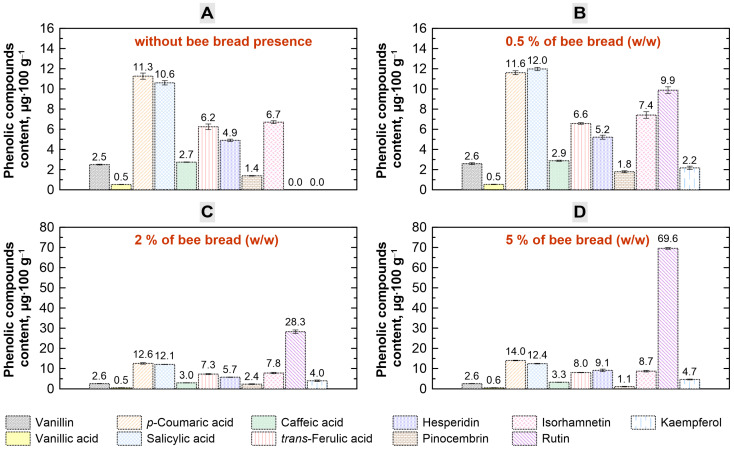
Changes in the phenolic compound contents in the honeydew honey in the presence of multifloral bee bread. (**A**) Phenolic compounds detected in the sample without the addition of bee bread. (**B**) Phenolic compounds detected in the sample with a 0.5% addition (*w*/*w*) of bee bread. (**C**) Phenolic compounds detected in the sample with a 2% addition (*w*/*w*) of bee bread. (**D**) Phenolic compounds detected in the sample with a 5% addition (*w*/*w*) of bee bread.

**Figure 2:**
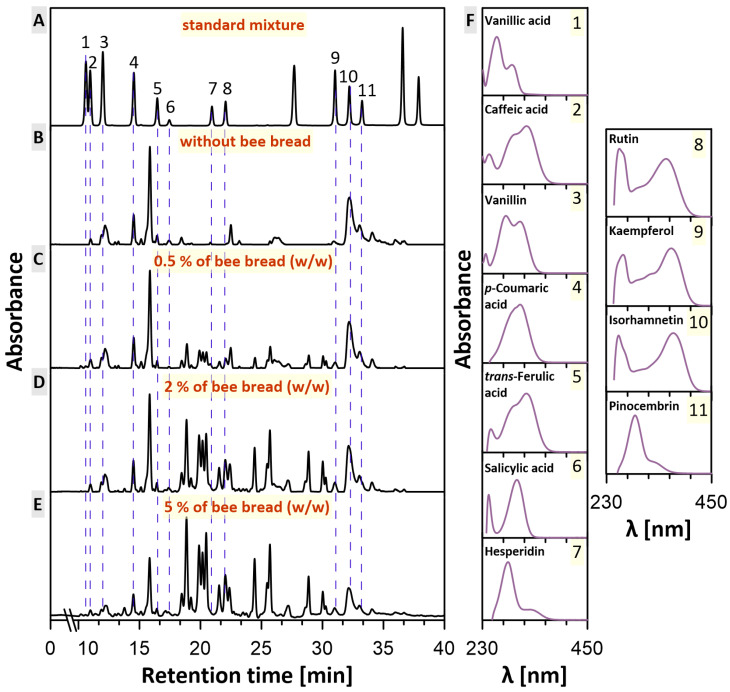
HPLC analysis of phenolic compounds. (**A**) Chromatogram of phenolic compounds in the standard mixture. (**B**–**E**) Chromatograms of phenolic compounds in the honeydew honey in the absence and presence of multifloral bee bread. (**F**) Absorption spectra from the diode array detection systems representing the elution bands 1–11 numbered in (**A**). The spectra were acquired in the course of the experiment presented in (**A**). Chromatograms are based on the absorbance level at 270 nm (to detect vanillic acid). Presented chromatograms and spectra are normalized to the most intense peak.

After the initial characterization of the starting products, honey and bee bread mixtures were prepared by adding the ground bee bread to honey at the levels of 0.5, 2 and 5% (*w*/*w*). Then, the resulting mixtures were tested for improved health-promoting properties. In particular, the profiles of phenolic compounds present in the individual samples in the absence and presence of bee bread were determined and are plotted in Figure 1. As can be seen, in all samples, the aldehyde called vanillin, the following phenolic acids—vanillic acid, *p*-coumaric acid, salicylic acid, caffeic acid and *trans*-ferulic acid—and the following flavonoids—hesperidin, pinocembrin and isorhamnetin—were marked. All of these phenolic acids and flavonoids have previously been detected in honeydew honey. Vanillic acid was found, for instance, in Turkish and Czech honeydew honey, *p*-coumaric acid in Polish, Turkish and Czech honeydew honey, salicylic acid in Czech, Croatian and Brazilian honeydew honey, caffeic acid in Polish, Turkish, Italian and Czech honeydew honey, *trans*-ferulic acid in Polish, Turkish and Czech honeydew honey, hesperidin in Brazilian honeydew honey, pinocembrin in Turkish, Czech and Spanish honeydew honey and isorhamnetin in Brazilian honeydew honey [24,25,26,27,28,29,30,31]. However, vanillin was found in acacia, buckwheat, fir, heather, linden, rape and thyme honeys [32]. In our study, the amounts of the separate phenols changed only slightly in the presence of bee bread. Generally, the higher the bee bread concentration, the more of these phenolic compounds by a trifle. Even so, it should be noted that after bee bread addition, the samples contained two additional flavonoids that were not present in honey—rutin and kaempferol—and the amount of rutin significantly increased with an increase in the concentration of bee bread used. Both of these compounds are known for their specific antioxidant, anticancer, anti-inflammatory and antidiabetic properties [33,34,35,36]. The antidiabetic effect of rutin had already been observed in 1993, when the administration of rutin in streptozotocin-induced diabetic rats resulted in a decrease in plasma glucose, an augmentation in insulin levels and a restoration of glycogen content and glycolytic enzymes [37]. In turn, in the case of kaempferol, it is believed that some kaempferol glycosides and several kaempferol-containing plants not only have antidiabetic effects but also can prevent diabetic complications [38]. Chromatograms obtained during the analysis of phenolic compounds along with the absorption spectra from the diode array detection system representing the subsequent elution bands are presented in Figure 2. The compounds were identified by comparing their retention times and spectra with reference standards.

**Figure 3:**
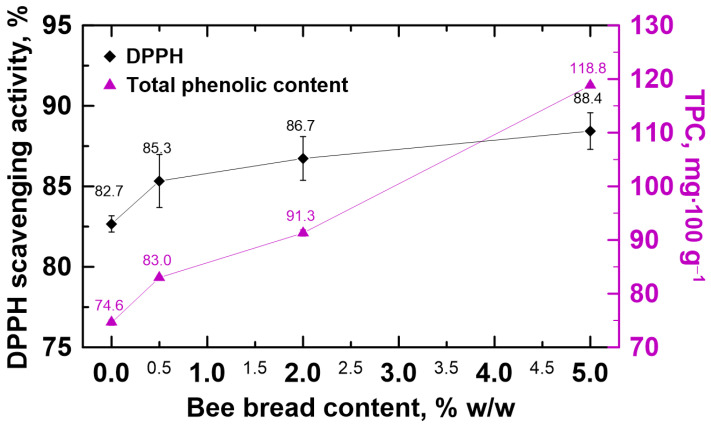
Changes in the DPPH radical scavenging activity and total phenolic content (TPC) in the honeydew honey in the presence of multifloral bee bread.

The honeydew honey supplemented with bee bread was also studied for DPPH radical scavenging activity and the total content of phenolic compounds (see Figure 3). The observed activity against the DPPH radical and the total content of phenolic compounds in pure honeydew honey were 82.7% and 74.6 mg·100 g^−1^, respectively. Similar findings were previously reported in Poland [10]. The results obtained after bee bread addition proved that the bee bread positively affected the antioxidant properties of honeydew honey. This is directly evident in the observed growth in radical scavenging activity and in the increase in the total content of phenolic compounds. As expected, the lowest addition of bee bread caused the smallest changes in the tested properties, while the highest addition caused the greatest changes. Interestingly, bee bread addition at a level as low as 0.5% (*w*/*w*) turned out to have an observable effect on the antioxidant properties of honeydew honey. The antioxidant activity of honey is highly correlated with phenolic compound presence [39]. On the other hand, honeydew honeys present high contents of minerals, proteins, organic acids, enzymes and amino acids, and these components are also known as antioxidant agents [23,39]. Although the exact mode of antioxidant action is unknown, there are several possible mechanisms involved in the antioxidant effects of honey [39,40,41]. The proposed mechanisms include free radical scavenging, the regulation of the expression of genes involved in reducing oxidative stress, the stimulation of the secretion of antioxidant enzymes, hydrogen donation, metallic ion chelation, flavonoid substrate action for hydroxyl, and superoxide radical actions [39,41].

**Figure 4:**
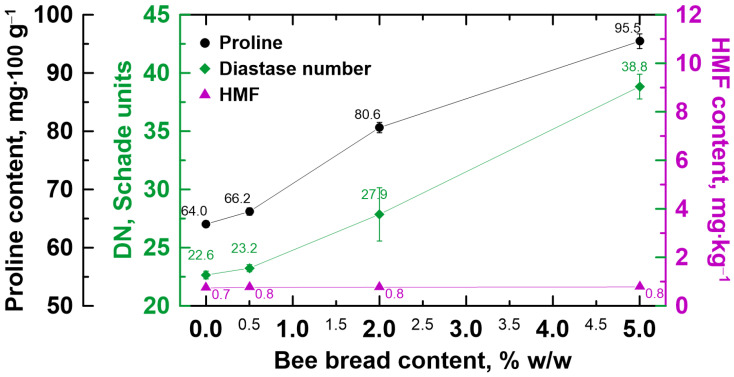
Changes in proline content, diastase number (DN) and hydroxymethylfurfural (HMF) content in the honeydew honey in the presence of multifloral bee bread.

Furthermore, the enrichment of honeydew honey with bee bread caused the desired increase in proline content and diastase activity and fortunately did not change the amount of harmful HMF (see Figure 4). The initial values were 64.0 mg·100 g^−1^, 22.6 Schade units and 0.7 mg·kg^−1^ for proline, DN and HMF, respectively, and were in good agreement with the literature data [21,23]. Proline is the dominant free amino acid found in honey, is a measure of total amino acid levels and contributes to the antioxidant activity of honey [42,43]. In addition, it is crucial for protein synthesis and cell growth, and plays an important role in other biological processes such as osmoregulation, redox signaling, unfolded protein responses, protein stability, cellular bioenergetics and stress resistance [44]. Studies indicate that it may also be a precursor of the biosynthesis of secondary metabolites with antibacterial or antifungal properties [44]. Diastase (α-amylase) is one of the main enzymes found in honey, and its activity level is considered an indicator of honey freshness and its proper processing [45]. HMF, in turn, is a furanic compound formed during the acidification or heating of products containing sugar, which determines the quality of honey [46]. Studies indicate its hepatocarcinogenic, genotoxic and cytotoxic effects [47,48]. It is worth mentioning that the international regulations only specify the minimum proline content and DN that should characterize honey; they do not specify the maximum value, which means that the more proline there is and the greater the diastase activity is, the better [20]. On the contrary, the HMF content should not be greater than 40 mg·kg^−1^ [20].

Finally, it should be added that the honeydew honey–bee bread mixture was tested not only for its health-promoting properties but also for its free acidity, pH, sugar profiles and water content. The free acidity and pH of honeydew honey changed after the bee bread addition from 23.6 to 42.3 mval·kg^−1^ and from 5.0 to 4.7, respectively. Although the change in the free acidity was significant, the overall physicochemical value of the honeydew honey was not reduced, as the results obtained even for the highest concentration of bee bread did not exceed the accepted standards [20]. The sugar profiles and water content results remained unchanged regardless of whether bee bread was present in the sample or not.

## 3. Materials and Methods

### 3.1. Chemicals

Glycerol (pure per analytical grade, ppa grade), gelatine (ppa grade), sodium hydroxide in a 0.1 M analytical weighed amount, sodium acetate trihydrate (ppa grade), potassium hexacyanoferrate (II) trihydrate (ppa grade), zinc acetate dihydrate (ppa grade), sodium carbonate (ppa grade), ethanol (≥96.0%) and Folin–Ciocalteu’s reagent were purchased from Chempur (Piekary Śląskie, Poland). The Phadebas Honey Diastase Test tablets were bought from Magle Life Sciences (Malmö, Sweden), and glacial acetic acid (ppa grade) and formic acid (≥98.0%) were bought from Pol-Aura (Zawroty, Poland). Methanol and isopropanol were obtained from J.T. Baker (Gliwice, Poland) and Avantor (Gliwice, Poland), respectively, and were of HPLC grade. Proline (≥99.5%), ninhydrin (ACS reagent), 2-methoxyethanol (≥99.8%), hydroxymethylfurfural (≥99.0%), vanillin (≥99.0%), *p*-coumaric acid (≥98.0%), salicylic acid (≥99.0%), quercetin (≥95.0%), kaempferol (≥97.0%), isorhamnetin (≥95.0%), acacetin (≥97.0%), caffeic acid (≥98.0%), rutin trihydrate (≥90.0%), hesperetin (≥95.0%), pinocembrin (≥95.0%), chrysin (≥98.0%), *trans*-ferulic acid (≥99.0%), vanillic acid (≥97.0%), hesperidin (≥80.0%), gallic acid (≥97.5%) and DPPH (2,2-difenylo-1-pikrylohydrazyl, purity ≥ 90.0%) were bought from Merck (Darmstadt, Germany). For the analyses, distilled or ultrapure water from the Milli-Q system (Merck, Germany, resistivity of 18.3 MΩ cm) was used.

### 3.2. Samples

The study included a single honeydew honey from the Island Beskid mountain range (part of the Western Beskids located between the Raba and Sądecka valleys, southern Poland) and multifloral bee bread donated by the Apiculture Department of the National Institute of Horticultural Research. Before research, the honey variety and the botanical origin of the bee bread were confirmed using conductometric and melissopalynological analyses, respectively. The honey samples were supplemented with bee bread at the levels of 0.5, 2 and 5% per mass of honey and were stored at room temperature (23 °C) in glass containers until analysis. Prior to usage, the bee bread was thoroughly dried and stored in a tightly closed container in a frozen state (−20 °C) to prevent microbial growth. Microscopic examination of the bee bread sediment after thawing did not reveal any undesirable contamination.

### 3.3. Conductometric Analysis

Electrical conductivity was determined according to the method developed by the International Honey Commission [49]. In brief, the conductivity of a solution containing 20 g of honey (dry weight basis) in 100 mL of distilled water was measured at 20 °C and expressed in mS·cm^−1^. Three repetitions were made, and the final result is presented as the mean ± standard deviation (SD).

### 3.4. Melissopalynological Analysis

Pollen analysis of the bee bread sample was performed as follows: 10 g of bee bread was dissolved in 50 mL of distilled water and left overnight to soak. Then, after thorough mixing, 20 μL of bee bread suspension was placed on a microscope slide. Next, the slide was dried and secured with glycerogelatin and a coverslip. The material was analyzed using an Olympus CX33 microscope (Tokyo, Japan) at 400× magnification. At least 300 consecutive pollen grains of nectar-producing, wind-pollinated and non-nectar-producing plants were determined on each microscopic slide, and then, the percentage of each pollen grain was calculated. Two repetitions were made, and the final result is presented as the average of the two results and rounded to the whole unit (%).

### 3.5. Phenolic Compound Analysis

The determination of individual phenolic compounds was carried out using high-performance liquid chromatography with a diode array detector (HPLC-DAD, Shimadzu, Tokyo, Japan), with slight modifications to the method developed for bee pollen samples presented earlier [50]. Before HPLC separation, the samples were subjected to a solid phase extraction. The procedure was as follows: 5 g of the sample was dissolved in acidified water (deionized water with 1% addition of formic acid), mixed for 30 min using a mechanical shaker at room temperature, and transferred to a 50 mL volumetric flask. After the addition of 0.5 mL of the Carrez’s I and Carrez’s II solutions, the flask was filled up to its volume with acidified water. Then, the obtained solution was filtered through soft filter paper, and 10 mL of the filtrate was loaded onto the C18-SPE column (5 mg/6 mL, Bakerbond, VWR International, Radnor, PA, USA), which was previously activated with 6 mL of methanol and 6 mL of acidified water. After the column was washed with 6 mL of acidified water, the phenolic compounds were eluted with 6 mL of methanol and collected into a 10 mL flask. The flask was filled up to its volume with methanol, and then, the solution was filtered through a 0.45 μm PTFE membrane filter. Finally, chromatographic analysis was performed with the application of a Shimadzu HPLC system (Tokyo, Japan) equipped with an SPD-M20A diode array detector and a reversed-phase C-18 Synergi Fusion-RP 80 Å column (Phenomenex, Torrance, CA, USA). The particle size of the column was 4 μm, the internal diameter was 4.6 mm and the length was 250 mm. The column was thermostated at 40 °C. The separation was performed under gradient conditions with two solvent systems (A—acidified water; B—methanol) using the following program: 84% solvent A and 16% solvent B at the initial stage, 10% solvent A and 90% solvent B at 50 min, and 84% solvent A and 16% solvent B at 51–62 min. The flow rate was 1 mL·min^−1^. For the quantitative analysis of phenols, the external standard method was applied. The determinations were carried out in triplicate.

### 3.6. DPPH Radical Scavenging Activity

The DPPH radical scavenging activity was determined according to the method described previously with some modifications [51]. Briefly, 2.5 g of the sample was weighed and transferred to a 50 mL volumetric flask, which was then filled up to its volume with methanol. The solution was then mixed and filtered through a soft filter paper. Next, 0.75 mL of the sample solution was mixed with 1.5 mL of DPPH solution in methanol (0.02 mg·mL^−1^). After 15 min of incubation at room temperature in the dark, the absorbance was measured using a Specord 200 spectrophotometer (Analytic Jena, Jena, Germany) at a wavelength of 517 nm. The final result is presented as a percentage of DPPH radical scavenging activity and is the average of three repetitions ± SD.

### 3.7. Total Phenolic Content Analysis

The total phenolic content was estimated using Folin–Ciocalteu’s method [52]. In the first step, 5 g of the sample was dissolved in ethanol–water solution (1:1, *v*:*v*), transferred to a 50 mL volumetric flask and combined with 0.5 mL of Carrez’s I and Carrez’s II reagents. Then, the flask was filled up to its volume with ethanol–water solution (1:1, *v*:*v*) and placed in a mechanical shaker for 30 min. After being filtered through a soft filter paper, 0.5 mL of the solution was collected and mixed with 2.5 mL of 10% Folin–Ciocalteu’s reagent. After 3 min of mixing, 2 mL of sodium carbonate solution (0.7 M) was added. The obtained mixture was mixed again and kept in the dark for 2 h at room temperature, after which the absorbance was read at a wavelength of 760 nm using a Specord 200 spectrophotometer (Analytic Jena, Germany). Gallic acid was used to calculate the standard curve, and the results are expressed as mg of gallic acid equivalents per 100 g of sample. The measurements were triplicated.

### 3.8. Proline, Diastase and Hydroxymethylfurfural Analysis

The proline content, diastase number and hydroxymethylfurfural content were also determined according to the methods developed by the International Honey Commission [49]. For the proline content analysis, 2.5 g of the sample was weighed and dissolved in 50 mL of water. Then, 0.5 mL of this solution was collected into three test tubes, and to each test tube, 0.25 mL of formic acid and l ml of 3% ninhydrin solution in 2-methoxyethanol were added. After vigorous mixing, the tubes were placed in a boiling water bath for 15 min. Next, the solutions in the test tubes were cooled to about 22 °C, 5 mL of an aqueous isopropanol solution (1:1, *v*:*v*) was added and mixed, and the absorbance at the 520 nm wavelength was measured using a Specord 200 spectrophotometer (Analytic Jena, Germany). The final result is the average of three repetitions ± SD.

The diastatic activity was determined by the Phadebas method in which diastase activity is expressed as the diastase number and is reported in Schade units [49]. First, 1 g of the analyzed sample was weighed and transferred to a 100 mL volumetric flask, which was then filled up to its volume with 0.1 M acetate buffer at pH = 5.2. Five milliliters of the sample was then transferred to the test tube and placed in a water bath at 40 °C for 15 min, and next, a Phadebas tablet was added to the solution. The solution was mixed and heated again in a water bath (40 °C) for 30 min. After this time, 1 mL of 0.5 M sodium hydroxide solution was added, and then, the solution was filtered through a filter paper (φ = 70 mm). Finally, the absorbance at the 620 nm wavelength was measured using a Specord 200 spectrophotometer (Analytic Jena, Germany). The measurements were triplicated, and the final result is their average ± SD.

The HMF content was determined chromatographically with the application of a Knauer HPLC system equipped with a UV K-2501 detector and a reversed-phase C-18 Vertex Plus Eurospher column (BGB Analytik Vertrieb GmbH, Rheinfelden, Germany), analogously to the method described previously [53]. The particle size of the column was 5 μm, the internal diameter was 4 mm and the length was 250 mm. The column and detector were thermostated at 30 °C. As a mobile phase, a mixture of methanol and deionized water (10:90, *v*/*v*) was used, and the flow rate was 1 mL·min^−1^. For the quantitative analysis of HMF, the external standard method was applied. The determinations were carried out in triplicate. Samples were prepared based on the European Honey Commission’s procedure [49].

## 4. Conclusions

In this study, honeydew honey samples enriched with multifloral bee bread were tested for their phenolic compound profile, DPPH radical scavenging activity, total phenolic content, diastase number and proline and HMF content. The obtained results indicated, firstly, that the bee bread addition provides two important flavonoids to honeydew honey, i.e., rutin and kaempferol. Both of these are known for their significant antioxidant, anticancer, anti-inflammatory and antidiabetic activities. Furthermore, the supplementation of honeydew honey with the bee bread caused the desired increase in radical scavenging activity and in the total content of phenolic compounds. Moreover, an increase in proline content and diastase activity was observed. However, the HMF content remained unchanged regardless of the absence or presence of bee bread. All of these findings prove the positive effect of bee bread addition on the properties of honeydew honey as well as highlight the health-promoting value of this mixture of bee products. The presented preliminary studies are characterized by a limited number of tested samples; however, the obtained results encourage an extension of the scope of the research with further samples in order to develop relevant statistical data. Moreover, this research motivates the expansion of bee product investigation to bring to light the natural product with the most beneficial functional properties for the targeted fight against various diseases, including lifestyle-related ones. For this purpose, it would be necessary to carefully examine a number of varietal honeys available on the market and analyze their additional properties, for example, antibacterial or antidiabetic properties.

## Figures and Tables

**Table 1:** Proportions of pollen grains identified in the bee bread sample.

**Pollen Type**	**Percentage**	* 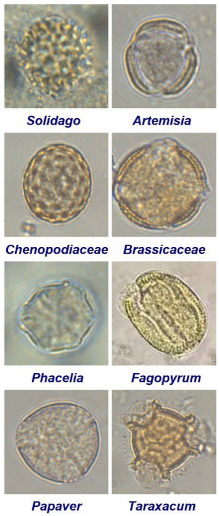 *
*Solidago*	40.7%	
*Artemisia*	25.7%	
*Chenopodiaceae*	9.5%	
*Brassicaceae*	7.5%	
*Phacelia*	5.8%	
*Fagopyrum*	3.0%	
*Papaver*	2.6%	
*Taraxacum*	1.2%	
*Tilia*	0.9%	
*Centaurea cyanus*	0.7%	
*Hypericum*	0.4%	
*Plantago*	0.4%	
*Zea mays*	0.4%	
*Viola tricolor*	0.3%	
*Cirsium*	0.3%	
Others	0.5%	

## Data Availability

The raw data supporting the conclusions of this article will be made available by the authors on request.
